# Hypoxia Impairs NK Cell Cytotoxicity through SHP-1-Mediated Attenuation of STAT3 and ERK Signaling Pathways

**DOI:** 10.1155/2020/4598476

**Published:** 2020-06-19

**Authors:** Rui Teng, Yanmeng Wang, Nan Lv, Dan Zhang, Ramone A. Williamson, Lei Lei, Ping Chen, Li Lei, Baiyan Wang, Jiaqi Fu, Xuna Liu, Aili He, Michael O'Dwyer, Jinsong Hu

**Affiliations:** ^1^Key Laboratory of Shaanxi Province for Craniofacial Precision Medicine Research, College of Stomatology, Xi'an Jiaotong University, 98 Xi Wu Road, Xi'an, 710004 Shaanxi, China; ^2^Department of Cell Biology and Genetics, Xi'an Jiaotong University Health Science Center, 76 Yanta West Road, Xi'an, 710061 Shaanxi, China; ^3^Department of Clinical Hematology, Second Affiliated Hospital, Xi'an Jiaotong University Health Science Center, 157 Xi Wu Road, Xi'an, 710004 Shaanxi, China; ^4^Biomedical Sciences, National University of Ireland Galway, Galway, Ireland

## Abstract

Natural killer (NK) cells are innate immune effectors with potent antitumor activity. However, tumor cells can create an immunosuppressive microenvironment to escape immune surveillance. Although accumulating evidence indicates that microenvironmental hypoxia plays an important role in favoring tumor development and immune evasion, it remains unclear by what means hypoxia directly impairs NK cell antitumor activity. In this study, we confirmed that hypoxic NK cells showed significantly lower cytotoxicity against tumor cells. Consistent with this finding, we found that the reduction in NK cell cytotoxicity resulting from hypoxia correlated to the lower expression of granzyme B, IFN-*γ*, and degranulation marker CD107a, as well as activating receptors including NKp30, NKp46, and NKG2D expressed on the surface of NK cells. More importantly, we further demonstrated that a reduction in the phosphorylation levels of ERK and STAT3 secondary to hypoxia was strongly associated with the attenuated NK cell cytotoxicity. Focusing on the mechanism responsible for reduced phosphorylation levels of ERK and STAT3, we reveal that the activation of protein tyrosine phosphatase SHP-1 (Src homology region 2 domain-containing phosphatase-1) following hypoxia might play an essential role in this process. By knocking down SHP-1 or blocking its activity using a specific inhibitor TPI-1, we were able to partially restore NK cell cytotoxicity under hypoxia. Taken together, we demonstrate that hypoxia could impair NK cell cytotoxicity by decreasing the phosphorylation levels of ERK and STAT3 in a SHP-1-dependent manner. Therefore, targeting SHP-1 could provide an approach to enhance NK cell-based tumor immunotherapy.

## 1. Introduction

Natural killer (NK) cells are cytotoxic innate lymphoid cells involved in the immune surveillance of tumors [1]. NK cells have a natural ability to kill tumor cells without any priming or prior activation, which is distinct from that of adaptive immune cells. Once the decision is made to kill, NK cells release cytotoxic granules containing granzyme and perforin to lyse the target cells. Activated NK cells can also upregulate death-inducing ligands such as Fas-L and TRAIL, which induce apoptosis in target cells via activation of the death receptor pathway [2]. In addition, NK cells can produce a variety of proinflammatory cytokines and chemokines to modulate innate and adaptive immune activities against tumors [3]. NK cell cytotoxicity is tightly regulated by a delicate balance between activating and inhibitory receptors [4]. Activating receptors, such as NKG2D, DNAM-1, and NK cytotoxicity receptors NKp30, NKp44, and NKp46 can recognize specific stress-induced ligands expressed on the surface of tumor cells, thus enhancing NK cell cytotoxicity [5]. On the other hand, the recognition of cognate major histocompatibility complex (MHC) class I molecules by killer cell immunoglobulin-like inhibitory receptors (KIRs) is important in suppressing the cytotoxic activity of NK cells against normal healthy cells [6, 7]. Loss of MHC I expression by tumor cells removes this inhibition, leading to enhanced NK cytotoxicity. Apart from the KIRs, a variety of other inhibitory receptors have been described, which may impact on NK cytotoxicity, including NKG2A, PD-1, T cell immunoreceptor with Ig and ITIM domains (TIGIT), CD96, Siglec-7, and Siglec-9 [8–11].

Although NK-cell-based immunotherapy is emerging as a promising approach to treat tumors, there remains a downside as tumors develop various strategies to evade NK cell attack or to impair the activity and function of NK cells [12]. In this regard, the failure of immune surveillance may be partially caused by the emergence of tumor escape variants due to sustained immunological pressure on tumor cells [13]. Alternatively, accumulating evidence also suggests that the major mechanisms of tumor escape from NK cell-mediated killing are tightly associated with the suppressive tumor microenvironment (TME) [14]. Tumor cells exploit cellular and noncellular TMEs to avoid recognition and destruction by the immune system. As a hallmark of solid tumors, it is well established that tumor hypoxia is a key factor regulating the loss of immune reactivity either by decreasing tumor cell sensitivity to cytotoxic immune effectors or by promoting immunosuppressive mechanisms [15–17]. More recently, hypoxia has been demonstrated to be a prevalent feature of the bone marrow microenvironment in acute myeloid leukemia and multiple myeloma, but not the normal healthy bone marrow [18–21]. Focusing on the mechanisms of hypoxia-mediated immune escape in cancer, previous studies mainly focused on the changes in tumors, for instance, hypoxia-induced release of immunosuppressive molecules or expression of immune checkpoint molecules. Little is known of the direct consequences of hypoxia on NK cell function [22–24].

In this study, focusing on the roles of hypoxia in NK cell-mediated immune surveillance in blood malignancies, we investigated the effects of hypoxia on NK cell cytotoxicity machinery and the underlying mechanisms. Our findings demonstrate a previously unknown role for hypoxia in the dysfunction of NK cell-mediated tumor surveillance and suggest that SHP-1 may represent a novel target for preserving NK cell function in cancer patients and improving NK cell-based immunotherapy.

## 2. Materials and Methods

### 2.1. Antibodies and Reagents

Antibodies for Western blotting against phospho-Stat3 (#4113), Stat3 (#12640), Phospho-p44/42 MAPK (ERK1/2) (#9106), p44/p42 MAPK (ERK1/2) (#9102), Phospho-SHP-1 (#8849), Phospho-SHP-2 (#5431), SHP-1 (#3759), SHP-2 (#3397), HIF-1*α* (#14179), and *β*-actin (#58169) were bought from Cell Signaling Technology (Danvers, Massachusetts, USA). Peroxidase-conjugated goat anti-rabbit IgG (#111-035-003) or goat anti-mouse IgG (#115-005-003) was bought from Jackson ImmunoResearch (West Grove, Pennsylvania, USA). For flow cytometry analysis, Alexa Fluor 647-labeled antihuman perforin (#563576), V450-labeled CD3 (#560365), and PE-labeled CD56 (#555516) were purchased from BD Biosciences (San Jose, California, USA). FITC-labeled Annexin V (#640945), PE-labeled antihuman IFN-*γ* (#506506), antihuman/mouse granzyme B (#372207), APC-labeled antihuman NKp46 (#137607), antihuman NKp30 (#325209), antihuman NKG2D (#320808), antihuman CD2 (#300214), and antihuman CD107a (#12-1079-42) antibodies were purchased from Biolegend (San Diego, California, USA). Sytox® Green Dead Cell Stain (#S34860) was bought from Molecular Probes (Waltham, Massachusetts, USA). STAT3 inhibitor Cryptotanshinone (#35825-57-1) was bought from Selleck Chemicals (Pittsburgh, Pennsylvania, USA). SHP-1 inhibitor TPI-1 (#HY-100463), SHP-2 inhibitor SHP-099 (#HY-100388), and ERK inhibitor U0126 (#HY-12031) were bought from MedChemExpress (Monmouth Junction, New Jersey, USA).

### 2.2. Cell Lines and Culture

The NK cell line KHYG-1 was cultured in RPMI-1640 supplemented with 10% fetal bovine serum (FBS) (#04-001-1ACS, Biological Industries, Kibbutz Beit-Haemek, Israel), 10 ng/mL human IL-2 (#200-02, PeproTech, Rocky Hill, New Jersey, USA), 2 mM L-glutamine, 100 U/mL penicillin, and 100 *μ*g/mL streptomycin. The NK cell line NK92 was cultured in Dulbecco's modified Eagle's medium (DMEM) supplemented with 10% FBS, 10% horse serum, 10 ng/mL IL-2, 2 mM glutamine, 100 U/mL penicillin, and 100 *μ*g/mL streptomycin. The human multiple myeloma cell line MM.1S and leukemia cell line K562 were grown in RPMI-1640 supplemented with 10% FBS, 2 mM glutamine, 100 U/mL penicillin, and 100 *μ*g/mL streptomycin. All cells were maintained at 37°C in a humidified atmosphere containing 5% CO_2_. Normoxic or hypoxic cell culture conditions were obtained by culturing cells in a sealed incubator flushed with a mixture of 20% O_2_, 5% CO_2_, and 75% N_2_ or the mixture of 1% O_2_, 5% CO_2_, and 94% N_2_, respectively.

### 2.3. Human Primary NK Cell Enrichment and Activation

Primary NK cells were isolated from peripheral blood mononuclear cells of healthy human donors through an immunomagnetic negative selection strategy (EasySep Human NK cell Isolation Kit #17955, Stemcell Technologies, Cambridge, Massachusetts, USA) according to the manufacturer's protocol. Purity of the purified NK cell populations was determined by flow cytometry using fluorochrome-conjugated antibodies against CD3 and CD56. For short-term activation, purified NK cells (>90% pure) were resuspended in RPMI-1640 supplemented with 10% FBS, 5% human serum, 2 mM glutamine, 100 U/mL penicillin, and 100 *μ*g/mL streptomycin at a density of 3 × 10^6^ cells/mL and cultured overnight in the presence of IL-2 (10 ng/mL) under normoxic or hypoxic conditions as described above.

### 2.4. Flow Cytometry

The expression of NK cell cytotoxicity effector molecules and activating receptors was analyzed by flow cytometry. For membrane staining, 5 × 10^5^ cells were collected and washed with staining buffer (PBS containing 0.1% NaN_3_ and 0.1% BSA) three times. The cells were then incubated for 30 min on ice, according to the instructions provided with the respective antibodies. After washing 3 times, the cells were resuspended in 300 *μ*L staining buffer in the presence of Sytox Green or 7-AAD, which were used to gate out dead cells. Acquisition of 10,000 cells per reaction was performed using a CytoFLEX Cytometer (Beckman Coulter Life Sciences, Atlanta, Georgia, USA). Data were analyzed with Flowjo v7.6.2 (Tree Star, Ashland, Oregon, USA). For intracellular staining, 5 × 10^5^ cells were collected and fixed with 1 mL 1% paraformaldehyde in PBS for 15 min at room temperature. After washing 3 times with cell stain buffer, the fixed cells were then resuspended in 2 mL permeabilization buffer (0.1% saponin in cell staining buffer) and incubated for 30 min at room temperature. The cells were collected again by centrifugation and stained with the antibody at an optimal working concentration in permeabilization buffer for 15 min on ice. After washing three times with permeabilization buffer, the cells were resuspended cells in 300 *μ*L cell staining buffer for final flow cytometric analysis.

### 2.5. CD107a Degranulation Assay

Degranulation of cytotoxic contents from NK cells was measured by analysis of the degranulation marker CD107a by flow cytometry. Briefly, NK cells and tumor cells were individually preincubated for 14-16 h at 20% or 1% O_2_ and after that, combined at 1 : 1 (*E* : *T*) ratio (effector to target cell ratio) at either 20% or 1% O_2_ in 24-well plate. 5 *μ*L of APC-labeled anti-CD107a was added to the wells within 5-10 min after combining NK and tumor cells. Subsequently, Monensin (#554724) and GolgiPlug (#555029) (1 : 1000 dilution; BD Biosciences, San Jose, California, USA) were added. After a total incubation time of 4 h, the plate was placed on ice to stop the reaction. Cells were then harvested and analyzed using flow cytometry.

### 2.6. Flow Cytometric Cytotoxicity Assay

Prior to the assay, NK cells and tumor cells were individually preincubated for 24 h at 5% CO_2_ with 20% or 1% O_2_ first. NK and target cells were then incubated under comparable conditions in different *E* : *T* ratios in a 24 well plate. After the 4 h incubation, samples were harvested and washed followed by a combinational staining with CD2-APC and Annexin V-FITC as well as Sytox® Green, in which CD2 was used to distinguish effector from target cells, and target cell death was detected with Annexin V-FITC and Sytox® Green. A minimum of 10,000 target events were collected per sample, and the results were analyzed using Flowjo v7.6.2.

### 2.7. Western Blotting

For Western blotting, treated and untreated NK cells were lysed in buffer containing 50 mM Tris, 150 mM NaCl, 1% Triton X-100, 1% sodium deoxycholate, 0.1% SDS, and protease inhibitors on ice for 30 min. Lysates were centrifuged at 12,000 rpm for 15 min, and supernatants were collected. Protein concentration was determined by the BCA protein assay kit (#WB003, HEART Biotech, Xi'an, Shhanxi, China). Equal amounts of protein were loaded and separated on sodium dodecyl sulfate-polyacrylamide gel electrophoresis gel and transferred onto a PVDF membrane. After blocking for 1 h with 5% nonfat milk in PBS with 0.1% Tween-20 at room temperature, the membrane was incubated with primary antibody at 4°C overnight. Immunoblots were visualized using HRP-conjugated secondary antibodies and the ECL Western Blot Detection kit (#PH0353, Phygene Life Sciences, Fuzhou, China).

### 2.8. siRNA-Mediated Gene Silencing in NK Cells

Prior to siRNA transfection, KHYG-1 cells were washed in prewarmed Opti-MEM medium (#SH30265.01, Life Technologies, Carlsbad, California, USA) and resuspended in the same medium. Then, 10^6^ cells were electroporated with 2 *μ*g of siRNA in 100 *μ*L Opti-MEM medium in 0.2 cm cuvette with an electroporator CUY21EDIT (BEX Co. Ltd, Japan). The electroporation program was set as follows: PpV = 200 V, Pp on 10 ms, Pp off 10 ms, PdV = 25 V, Pd on 50 ms, Pd off 50 ms; Pd N = 10, capacity = 940 *μ*F, and exponential decay wave type. Following electroporation, cells were resuspended in 2 mL complete media and cultured in hypoxic condition (1% O_2_). 16-24 h after electroporation, the cells were used for Western blotting or killing assay. Transfection efficiency and viability were analyzed by flow cytometry 2-6 h after electroporation by quantitatively measuring the expression of fluorescein isothiocyanate- (FITC-) labeled siRNA and 7-AAD. SHP-1 mRNA was silenced by using a gene-specific siRNA pool (GenePharma, Shanghai, China) (see Supplementary Table 1).

### 2.9. Statistical Analysis

Statistical analyses were performed using the Prism software package 5.0 (GraphPad Software, San Diego, California, USA). Data are expressed as the mean ± SEM of at least three independent experiments. Statistical significance was evaluated by two-tailed paired Student's *t-*test. A ^∗^*P* < 0.05, ^∗∗^*P* < 0.01, or ^∗∗∗^*P* < 0.001 was considered statistically significant.

## 3. Results

### 3.1. Hypoxic NK Cells Show Decreased Cytotoxicity against Tumor Cells

**Figure 1:**
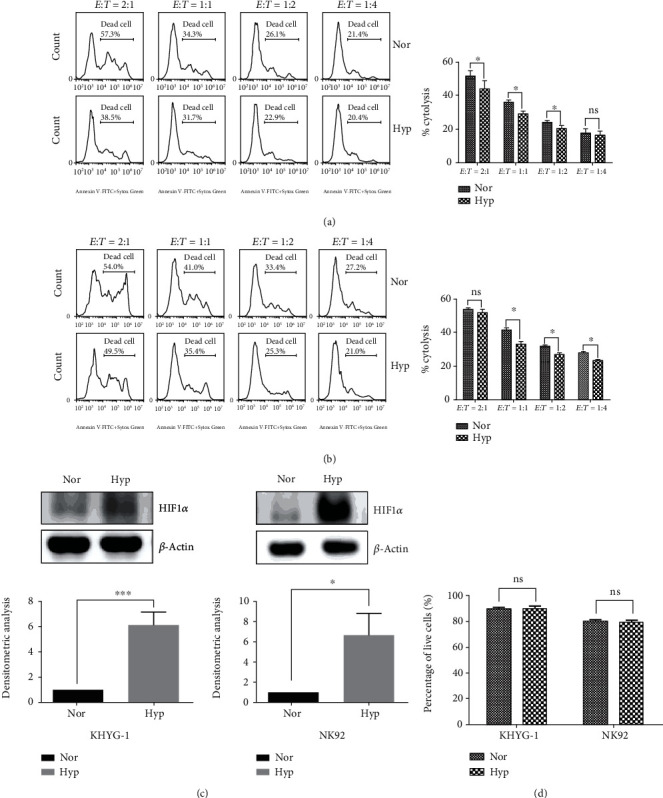
Hypoxic NK cells show lower cytotoxicity against tumor cells. (a, b) Flow cytometric analysis of KHYG-1 cells cytotoxicity against tumor cells. KHYG-1 cells were incubated with K562 (a) or MM.1S (b) tumor cells for 4 h at different *E* : *T* ratios after cultivation at normoxic (20% O_2_) and hypoxic (1% O_2_) conditions for 24 h. Left panel: a representation of results from three experiments; Right panel: statistical analysis showing the percentage of tumor cells killed by NK cells (*n* = 3, ^∗^*P* < 0.05). (c) Western blotting analysis of the effects of hypoxia on the expression of hypoxia marker HIF-1*α*. NK cells were cultured in 20% or 1% O_2_ for 24 h, and then Western blotting analysis was performed. Representative Western blot images are shown in the upper panel; the densitometric analysis is shown in bottom panel (*n* = 3, ^∗^*P* < 0.05, ∗∗∗*P* < 0.001). (d) Flow cytometric analysis of the effects of hypoxia on NK cell viability by performing Annexin V-FITC/7-AAD staining. NK cells were cultured in 20% or 1% O_2_ for 24 h, then flow cytometric staining was performed (*n* = 3, ns: no significance).

We first investigated whether hypoxia impairs NK cell-mediated lysis of tumor cells. To this end, KHYG-1 NK cells were cultured in the presence of IL-2 under hypoxic (1% O_2_) or normoxic (20% O_2_) conditions for 24 h and subsequently incubated with the hypoxic or normoxic tumor cell lines K562 or MM.1S at different *E* : *T* ratios for another 4 h to evaluate the cytotoxicity by flow cytometry. As shown in Figures 1(a) and 1(b), it revealed that the NK cell cytotoxicity was significantly decreased by 1% compared to 20% O_2_. Meanwhile, we observed a marked accumulation of the hypoxia marker HIF-1*α* in hypoxic NK cells, whereas it was weakly expressed in normoxic NK cells monitored by Western blotting (Figure 1(c)). Moreover, we excluded the possibility that the decreased cytotoxicity in hypoxia was caused by reduced NK cell viability since we did not observe increased NK cell death by hypoxia (Figure 1(d)).

### 3.2. Hypoxia Decreases the Expression of Cytotoxic Effectors and Activating Receptors on NK Cells

**Figure 2:**
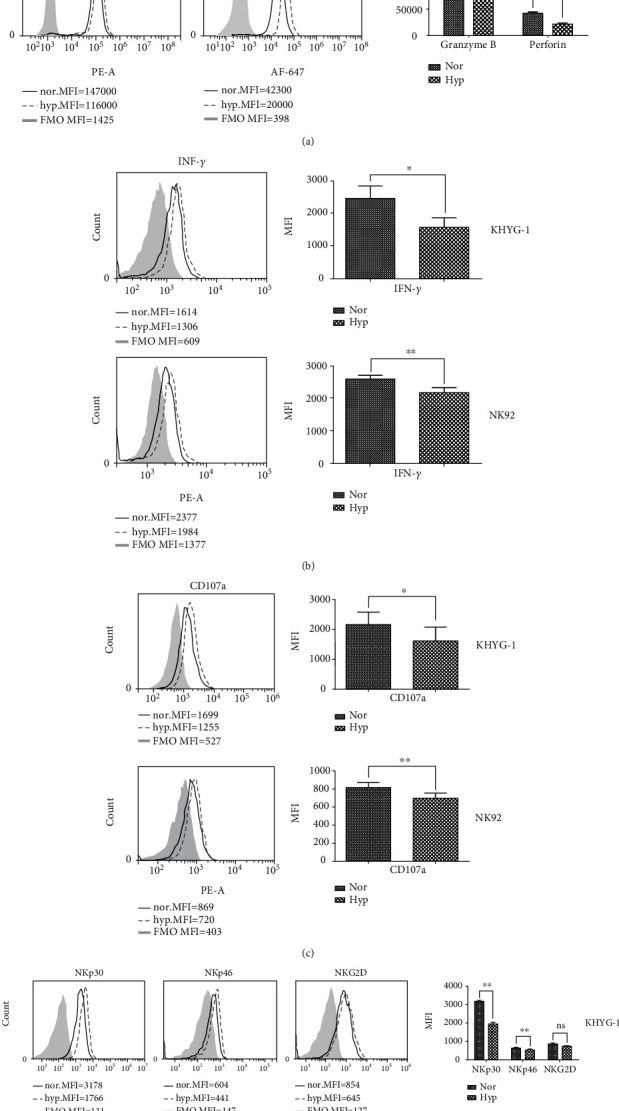
Hypoxia decreases the expression level of NK cells cytotoxicity related molecules. (a) Flow cytometric analysis of granzyme B and perforin expression in KHYG-1 (upper panel) and NK92 (lower panel) cells, respectively. KHYG-1 and NK92 were cultured in normoxic (20% O_2_) and hypoxic (1% O_2_) for 24 h, then intracellular staining was performed to analyze the expression of granzyme and perforin quantitatively. Left panel: histogram overlays display representative examples of granzyme B and perforin expression analyzed in normoxic and hypoxic cell samples compared to the fluorescence minus one (FMO) control; Right panel: statistical analysis of the flow cytometry data (*n* = 3, ^∗^*P* < 0.05, ^∗∗^*P* < 0.01). (b) Flow cytometric analysis of the intracellular level of IFN-*γ* in normoxic and hypoxic KHYG-1 and NK92 cells. Left panel: one of three representative flow cytometry results; Right panel: statistical analysis of the flow cytometry data (*n* = 3, ^∗^*P* < 0.05, ^∗∗^*P* < 0.01). (c) Flow cytometric analysis of the membrane staining of degranulation marker CD107a in normoxic and hypoxic KHYG-1 and NK92 cells. Left panel: one of three representative flow cytometry results; Right panel: statistical analysis of the flow cytometry data (*n* = 3, ^∗^*P* < 0.05, ^∗∗^*P* < 0.01). (d) Flow cytometric analysis of the membrane staining of activating receptor NKp30, NKp46, and NKG2D in normoxic and hypoxic KHYG-1 and NK92 cells. Left panel: one of three representative flow cytometry results; Right panel: statistical analysis of the flow cytometry data (*n* = 3, ^∗^*P* < 0.05, ^∗∗^*P* < 0.01).

To further explore how hypoxia reduces NK cell killing ability, we measured the expression level of the cytotoxic effectors granzyme B and perforin. As shown in Figure 2(a), hypoxia treatment led to decreased secretion of both granzyme B and perforin. Additionally, we observed a reduced expression of the cytokine IFN-*γ* in hypoxic NK cells compared to normoxic condition (Figure 2(b)). Importantly, CD107a, which is a degranulation marker of natural killer cell activity, was also diminished by hypoxia (Figure 2(c)). Given that a range of receptors that can trigger cytolytic programs, as well as cytokine or chemokine secretion tightly regulates NK cell function, we next evaluated the effects of hypoxia on the expression of the main receptors capable of triggering cytolytic activity. Surface expression of the activating receptors, including NKp46, NKp30, and NKG2D, was measured by flow cytometry on both normoxic and hypoxic NK cells. As shown in Figure 2(d), it confirmed that hypoxia could decrease the expression of activating receptors on the NK cell surface.

### 3.3. Hypoxia Attenuates ERK and STAT3-Mediated NK Activation

**Figure 3:**
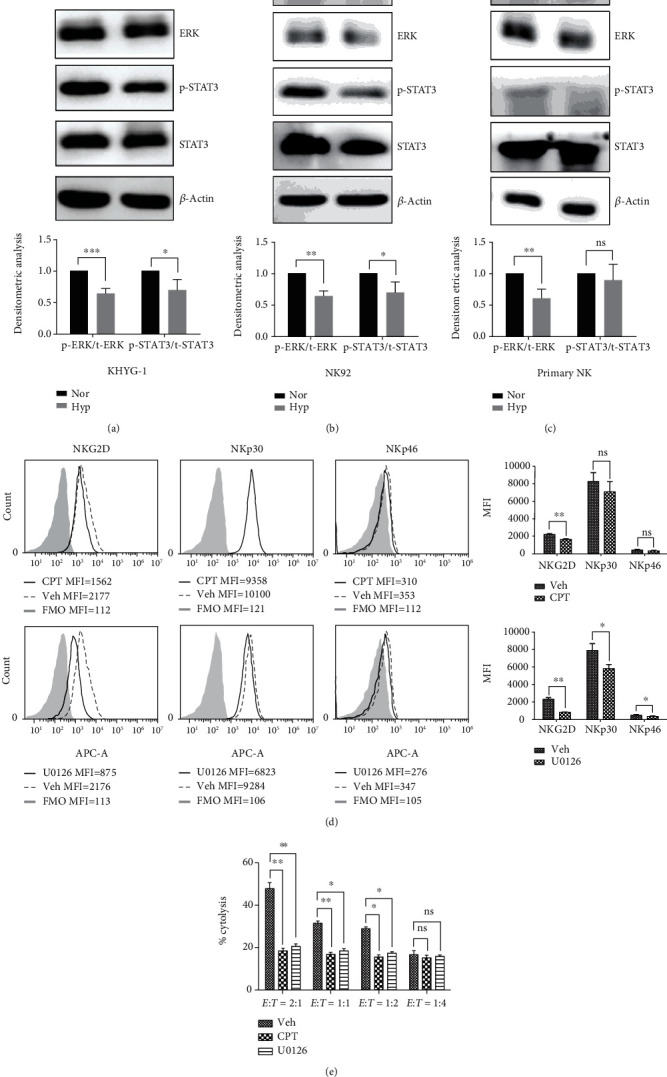
Hypoxia diminishes the phosphorylation level of ERK and STAT3. (a–c) Western blotting analysis shows the expression levels of the phosphorylated ERK and STAT3 in KHYG-1 (a), NK92 (b), and primary NK cells (c), respectively. Upper panel: representative Western blot images are shown from three independent experiments; lower panel: densitometric analysis of the p-ERK/ERK and p-STAT3/STAT3 band gray optical density ratios (*n* = 3, ^∗^*P* < 0.05, ^∗∗∗^*P* < 0.001). (d) Inhibition of ERK and STAT3 decreases the expression of activating receptors on the NK cell surface. Representative flow cytometry results show the effects of STAT3 inhibitor cryptotanshinone (CPT) (upper panel) and ERK inhibitor U0126 (lower panel) on the expression of activating receptors on the NK cell surface. Left panel: one of three representative flow cytometry results; Right panel: statistical analysis of the flow cytometry data (*n* = 3, ^∗^*P* < 0.05, ^∗∗^*P* < 0.01). KHYG-1 and NK92 cells were treated with vehicle, 10 *μ*M ERK inhibitor U0126, and 10 *μ*M STAT3 inhibitor CPT for 24 h. (e) Inhibition of ERK and STAT3 decreases NK cell cytotoxicity. Statistical analysis showing the effects of ERK and STAT3 inhibition on NK cells cytotoxicity against K562 cells (*n* = 3, ^∗^*P* < 0.05, ^∗∗^*P* < 0.01). KHYG-1 cells were pretreated with 10 *μ*M U0126 and 10 *μ*M CPT for 6 h and then incubated with K562 at different *E* : *T* ratios for 4 h.

It is known that intracellular signals activating NK cell cytotoxic activity are propagated primarily through protein phosphorylation of ERK (extracellular signal-regulated kinase) and STAT3 (signal transducer and activator of transcription 3) [25, 26]. Therefore, we further investigated whether hypoxia could affect the activation of ERK and STAT3 and revealed that hypoxia markedly diminished the phosphorylation level at the tyrosine sites of ERK and STAT3 in the two NK cell lines and primary NK cells (Figures 3(a)–3(c)). To further validate the effects of the phosphorylation of ERK and STAT3 on the expression of activating receptors and NK cytotoxicity under hypoxic conditions, we used specific small molecule inhibitors U0126 and cryptotanshinone to block ERK and STAT3 signaling, respectively. As shown in Figure 3(d), inhibition of ERK and STAT3 significantly reduced the expression of activating receptors, including NKp30 and NKG2D. Importantly, we found that inhibition of ERK or STAT3 resulted in significantly impaired cytotoxicity against tumor cells (Figure 3(e)).

### 3.4. Hypoxia-Decreased Phosphorylation Level of STAT3 and ERK Was Mediated by the Activation of Protein Tyrosine Phosphatase SHP-1 Rather than SHP-2

**Figure 4:**
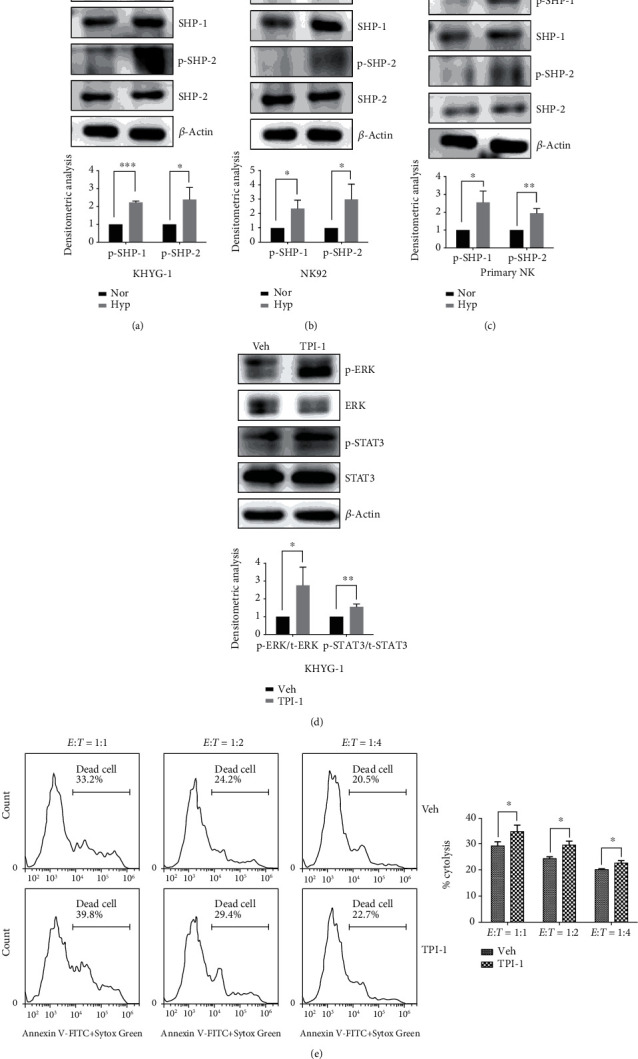
Hypoxia activates SHP-1 and SHP-2 in NK cells. (a–c) Western blotting analysis shows SHP-1 and SHP-2 expression in normoxic (20% O_2_) and hypoxic (1% O_2_) KHYG-1 (a), NK92 (b) cells, and primary NK cells (c), respectively. (d) Western blotting analysis shows the effects of SHP-1 inhibitor TPI-1 on the phosphorylation of ERK and STAT3. Hypoxic KYHG-1 cells were pretreated with 5 *μ*M TPI-1 for 2 h, and then the cells were collected for Western blotting analysis. Representative Western blot images are shown in upper panel; the densitometric analysis is shown in lower panel (*n* = 3, ^∗^*P* < 0.05, ^∗∗^*P* < 0.01, ^∗∗∗^*P* < 0.001). (e) Flow cytometric analysis of the effects of TPI-1 on the NK cell cytotoxicity. Left panel: representative flow cytometry results of TPI-1 on the cytotoxicity of KHYG-1 cells. KHYG-1 cells were pretreated with 5 *μ*M TPI-1 for 2 h and then incubated with K562 cells at different *E* : *T* ratios for 4 h. Right panel: statistical analysis of the effects of TPI-1 on KHYG-1 cell cytotoxicity against K562 cells (*n* = 3, ^∗^*P* < 0.05).

**Figure 5:**
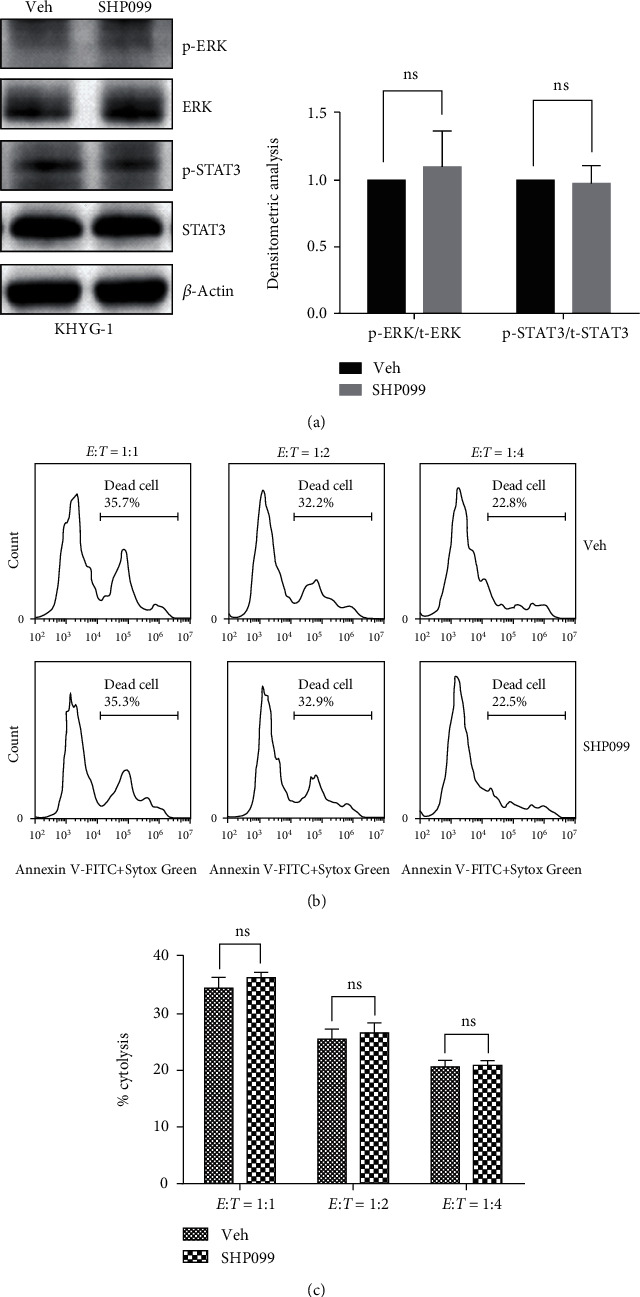
Inhibition of SHP-2 has no effect on NK cell cytotoxicity. (a) Western blotting shows the effects of SHP-2 inhibitor SHP099 on the phosphorylation of ERK and STAT3. Hypoxic KYHG-1 cells were pretreated with 5 *μ*M SHP099 for 2 h, and then the cells were collected for Western blotting analysis. Representative Western blot images are shown in left panel; the densitometric analysis is shown in right panel (*n* = 3, ^∗^*P* < 0.05, ^∗∗^*P* < 0.01, ^∗∗∗^*P* < 0.001). (b) Flow cytometric analysis shows the effect of SHP099 on the cytotoxicity of KHYG-1 cells. KHYG-1 cells were pretreated with 5 *μ*M SHP099 for 2 h, then incubated with K562 cells at different *E* : *T* ratios for 4 h. (c) Statistical analysis of the effects of SHP099 on KHYG-1 cell cytotoxicity against K562 cells (*n* = 3, ns: no significance).

Cell surface receptors harboring intracytoplasmic tyrosine-based activation motifs (ITAMs) or intracytoplasmic tyrosine-based inhibitory motifs (ITIMs) are often phosphorylated by Src family protein tyrosine kinase (PTK), which in turn creates docking sites for the protein tyrosine phosphatases SHP-1 and SHP-2. Recruitment and activation of the SHP-1 and/or SHP-2 have been demonstrated to be a dominant inhibitory mechanism to prevent the induction of the stimulatory signaling cascade [27, 28]. In this regard, we further investigated whether SHP-1 and SHP-2 were involved in the decrease of ERK and STAT3 phosphorylation by hypoxia. As shown in Figures 4(a)–4(c), hypoxia induced a significant increase in the phosphorylation of SHP-1 and SHP-2 in the two NK cell lines and primary NK cells. When using a specific SHP-1 inhibitor TPI-1, we observed it could reverse the decrease of the phosphorylation of both ERK and STAT3 (Figure 4(d)). Moreover, we also observed that pretreatment with the p-SHP1 inhibitor TPI-1 could restore the NK cell cytotoxicity under hypoxia (Figure 4(e)). However, we did not observe the same effects when using a specific SHP-2 inhibitor SHP099, which had no effect on the phosphorylation levels of ERK and STAT3, or NK cell cytotoxicity (Figure 5).

### 3.5. Knockdown of SHP-1 Rescues NK Cell Cytotoxicity in Hypoxia

**Figure 6:**
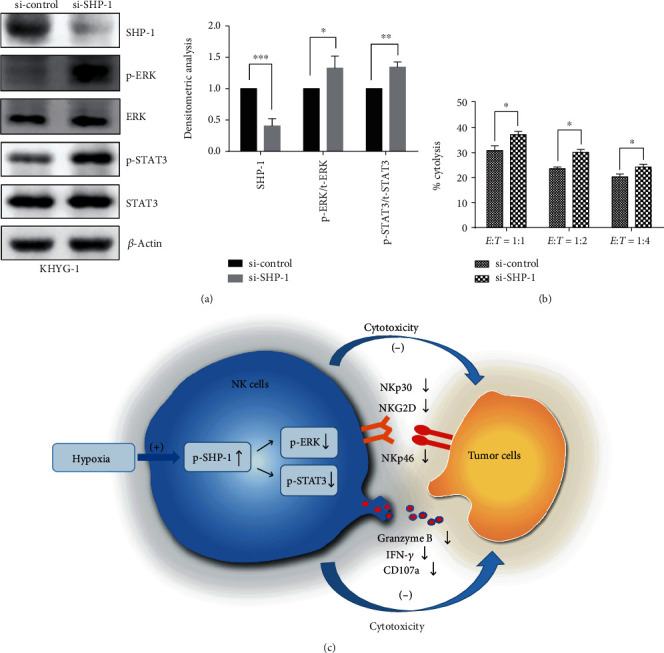
The effects of gene silencing SHP-1 on ERK and STAT3 signaling as well as NK cell cytotoxicity. (a) Western blotting analysis of the SHP-1, ERK, and STAT3 expressions in siRNA-mediated knockdown of SHP-1 in KHYG-1 cells. Representative Western blot images are shown in left panel; the densitometric analysis is shown in right panel (*n* = 3, ^∗^*P* < 0.05, ^∗∗^*P* < 0.01, ^∗∗∗^*P* < 0.001). (b) Statistical analysis of the effects of knocking down SHP-1 on NK cells cytotoxicity against K562 cells (*n* = 3, ^∗^*P* < 0.05). KHYG-1 cells were electroporated with 2 *μ*g siRNA and then cultured for 12-16 h in the RPMI 1640 growth medium containing IL-2. The electroporated cells were used for Western blotting or killing assay as previously mentioned. (c) A schematic diagram shows how hypoxia impairs NK cell cytotoxicity in a SHP-1-dependent manner.

To further validate the role of SHP-1 in regulating NK cell cytotoxicity, we silenced the gene expression of SHP-1 in KHYG-1 cells and confirmed that knockdown of SHP-1 could increase the phosphorylation level of ERK and STAT3 under hypoxia (Figure 6(a)). More importantly, we also confirmed that NK cells with SHP-1 silencing showed greater cytotoxicity against K562 cells than control NK cells under hypoxic conditions (Figure 6(b)).

## 4. Discussion

Accumulating evidence strongly suggests that hypoxia within the tumor microenvironment is likely to exert a negative effect on NK cell function. Within the tumor microenvironment, hypoxia exerts its effect on NK cell function via indirect effects on the tumor and its microenvironment, as well as the direct effect of hypoxia on NK cells. Since the former has already been studied in greater detail, the focus of this study was on the direct effect of hypoxia on NK cell function, independent of any tumor-related inhibition. Previous *in vitro* studies addressing this issue have shown conflicting results, which could reflect differences in experimental design, such as duration of hypoxic exposure and/or cytokine supplementation [17, 23]. We first demonstrated that hypoxia could directly impair NK cell cytotoxicity by decreasing the expression of activating receptors on the NK cell surface. More importantly, we revealed that impaired NK cell cytotoxicity secondary to hypoxia could be mediated by increased activation of the phosphatase SHP-1, which catalyzes the dephosphorylation at the tyrosine sites of ERK and STAT3, thereby attenuating NK cell activation signaling (Figure 6(c)).

NK cells use a combination of receptors and signaling pathways to protect the host against tumors [29]. NK cells express a variety of activating and inhibitory receptors to recognize cellular stress ligands as well as MHC I and related molecules, with the balance of activating and inhibitory signals determining NK cell responsiveness. Activating receptors initiate PTK-dependent signaling through noncovalent associations with transmembrane signaling adaptors that harbor intracytoplasmic ITAMs. On the other hand, inhibitory receptors such as KIRs, NKG2A, and Siglecs-7 and 9 contain ITIMs to inhibit NK-cell-mediated cytotoxicity [30]. Many of these receptors can signal through ITIM-dependent and ITIM-independent pathways to maintain a state of proper responsiveness. In this study, we found that hypoxic NK cells express a lower level of activating receptors, including NKp30, NKp46, and NKG2D. Our findings are in line with previous work also where decreased cell surface expression of activating receptors and CD16 was observed in association with hypoxia-induced impairment of NK cytotoxicity [31, 32]. However, control of NK cell cytotoxicity is not a simple balance between signals from activating and inhibitory receptors. NK cell function is also regulated by interaction with other immune cells as well as various soluble factors such as cytokines, chemokines, lactate, and hypoxia within the tumor microenvironment [29, 33]. Since IL-2 and IL-15 are known to play an important role in the proliferation, survival, and cytotoxicity of NK cells, exposure to these cytokines is frequently employed to enable significant expansion of the NK cell subpopulations *in vitro* and *ex vivo* [34, 35]. IL-2 and IL-15 activated signaling pathways including PI3K-AKT-mTOR, JAK-STAT, and MEK-ERK have been demonstrated to play pivotal roles in NK cell proliferation and activation [34–36]. In this regard, our observations suggest activation of both ERK and STAT3 is necessary for the activation of NK cells *in vitro*, especially following treatment with IL-2. Following inhibition of both ERK and STAT3, we observed a significant reduction in NK cell cytotoxicity along with a reduction in expression of activating receptors, suggesting an essential role of ERK and STAT3 signaling in regulating NK cytotoxicity. Of note, we also observed that hypoxia could decrease the phosphorylation of ERK and STAT3, in line with the lower cytotoxicity of hypoxic NK cells. Indeed, while IL-2 has been shown to improve the cytotoxicity of NK cells under hypoxia, cytotoxicity was still significantly inferior to that of IL-2 treated NK cells under normoxic conditions [31].

With respect to the mechanisms of hypoxia-mediated inhibition of ERK and STAT3, our findings suggest that the protein tyrosine phosphatase SHP-1 is a key player in this process. It is now accepted that SHP-1 and SHP-2 are recruited by inhibitory receptors to diminish ITAM associated tyrosine-phosphorylation [27, 28, 37–40]. SHP-1 and SHP-2 have been implicated in the regulation of a variety of tyrosine kinase-linked receptors, including cytokine and growth factor receptors, as well as ITAM-containing immune receptors. Several studies have shown that either SHP-1 or SHP-2 is involved in the downregulation of the phosphorylation of ERK or STAT3 by removing phosphates in different cell contexts [41–45], and the activity of SHP-1 and SHP-2 is tightly dependent on its phosphorylation at different tyrosine residues in carboxy-terminal [43, 46–49]. Concerning the roles of phosphatase SHP-1 and SHP-2 in NK cells, different studies have suggested that the expression of SHP-1 or SHP-2 may play opposite roles in regulating NK cell activity and function [28, 50, 51], suggesting that the roles of SHP-1 and SHP-2 in signaling are not redundant, although structurally similar [39, 52]. Herein, we demonstrated that while both SHP-1 and SHP-2 could be activated by hypoxia in NK cells, SHP-1 alone appeared to be involved in regulating the phosphorylation of ERK and STAT3, and therefore NK cell cytotoxicity. Collectively, our findings suggest a mechanism whereby hypoxia could dampen NK cell cytotoxicity via negative regulation of ERK and STAT3 in a SHP-1-dependent manner. Future studies will be required to unravel the molecular mechanisms influencing the hypoxic response in NK cells, including any role for HIF-1*α* and how this might relate to NK cytotoxicity.

In conclusion, we demonstrated that SHP-1 plays an important role in hypoxia-impaired NK cell cytotoxicity. Our finding supports that targeting SHP-1 may provide an important approach for improving NK cell-based tumor immunotherapy.

## Data Availability

The data used to support the findings of this study are available from the corresponding author upon request.

## Abbreviations

DMEM: Dulbecco's modified Eagle's medium
ERK: Extracellular signal-regulated kinase
*E* : *T* ratio: Effector to target cell ratio
FITC: Fluorescein isothiocyanate
ITAM: Intracytoplasmic tyrosine-based activation motifs
ITIM: Intracytoplasmic tyrosine-based inhibitory motifs
KIR: Killer cell immunoglobulin-like receptors
MHC: Major histocompatibility complex
NK: Natural killer
PTK: Protein tyrosine kinase
PTP: Protein tyrosine phosphatase
SHP-1: Src homology region 2 domain-containing phosphatase-1
SHP-2: Src homology region 2 domain-containing phosphatase-2
STAT3: Signal transducer and activator of transcription 3
TIGIT: T cell immunoreceptor with Ig and ITIM domains
TME: Tumor microenvironment.

## Supplementary Materials

Supplementary Materials Supplementary Table 1: siRNA sequences.
