# Corrigendum to “Hypoxia Impairs NK Cell Cytotoxicity through SHP-1-Mediated Attenuation of STAT3 and ERK Signaling Pathways”

**DOI:** 10.1155/2021/3625084

**Published:** 2021-02-25

**Authors:** Rui Teng, Yanmeng Wang, Nan Lv, Dan Zhang, Ramone A. Williamson, Lei Lei, Ping Chen, Li Lei, Baiyan Wang, Jiaqi Fu, Xuna Liu, Aili He, Jinsong Hu, Michael O'Dwyer

**Affiliations:** ^1^Key Laboratory of Shaanxi Province for Craniofacial Precision Medicine Research, College of Stomatology, Xi'an Jiaotong University, 98 Xi Wu Road, Xi'an, 710004 Shaanxi, China; ^2^Department of Cell Biology and Genetics, Xi'an Jiaotong University Health Science Center, 76 Yanta West Road, Xi'an, 710061 Shaanxi, China; ^3^Department of Clinical Hematology, Second Affiliated Hospital, Xi'an Jiaotong University Health Science Center, 157 Xi Wu Road, Xi'an, 710004 Shaanxi, China; ^4^Biomedical Sciences, National University of Ireland Galway, Galway, Ireland

In the article titled “Hypoxia Impairs NK Cell Cytotoxicity through SHP-1-Mediated Attenuation of STAT3 and ERK Signaling Pathways” [1], the order of the corresponding authors was incorrect in the original article and the corrected author list is shown above.
