# Supplementary material for: Hypoxia Impairs NK Cell Cytotoxicity through SHP-1-Mediated Attenuation of STAT3 and ERK Signaling Pathways
Source: J Immunol Res. 2020 Jun 19;2020:4598476. doi: 10.1155/2020/4598476 (PMC7584946; doi:10.1155/2020/4598476)
Supplement: Supplementary Materials — Supplementary Table 1: siRNA sequences. [file 4598476.f1.docx]

| **Gene** | **Sense** | **Antisense** |
| --- | --- | --- |
| **SHP-1** | 5’-GAGCAUGACACAACCGAAUTT-3’ | 5’-AUUCGGUUGUGUCAUGCUCTT-3’ |
|  | 5’-GCGCAGUACAAGUUCAUCUTT-3’ | 5’-AGAUGAACUUGUACUGCGCTT-3’ |
|  | 5’-GGGAUUUCUAUGACCUGUATT-3’ | 5’-UACAGGUCAUAGAAAUCCCTT-3’ |
| **Non-targeting control** | 5’-UUCUCCGAACGUGUCACGUTT-3’ | 5’-ACGUGACACGUUCGGAGAATT-3’ |

**Supplementary Table 1. siRNA sequences.**
